# Bonding super translucent multilayered monolithic zirconia to different foundation materials: an invitro study

**DOI:** 10.1186/s12903-024-05244-z

**Published:** 2024-12-18

**Authors:** Noha Essam, Shaimaa Ahmed Abo-Elfarag, Ahmed Attia

**Affiliations:** Department of Fixed Prosthodontics, Faculty of Dentistry, Mansoura University, Mansoura, Dakahlia Governorate Egypt

**Keywords:** Super translucent, Zirconia, Bond strength, Surface treatment, Foundation materials

## Abstract

**Background:**

The objective of this study was to investigate the effect of bonded substrate, zirconia surface conditioning and the interaction between them on the shear bond strength of monolithic zirconia.

**Methods:**

Forty-eight monolithic zirconia discs were CAD-CAM fabricated and divided into two groups according to surface treatment either as milled and universal primer application (Monobond N, Ivoclar-Vivadent) (P) or sandblasting then universal primer application (Monobond N) (SP). Each main group was further divided into three test groups according to the bonded substrate: dentin (DSP, DP), composite (CSP, CP) or resin modified glass ionomer (RMGI) (GSP, GP). Adhesive resin cement (Multilinik automix, Ivoclar-Vivadent) was used for bonding. Specimens were stored in water bath for six months before thermal cycling for 10,000 cycles to mimic intra oral condition. All specimens underwent shear bond strength test **(SBS)** using a universal testing machine. Two and one-way ANOVA and Bonferroni Post Hoc tests were used for statistical analyses.

**Results:**

The means ± SD SBS of all test groups were recorded in (MPa). DSP group showed the highest mean SBS (22.65 ± 2.0) followed by DP group (18.61 ± 2.55). Meanwhile, GSP and GP groups showed the lowest mean SBS (4.77 ± 0.09, 4.57 ± 0.73 respectively).

**Conclusion:**

Sandblasting with priming is recommended as a monolithic zirconia surface treatment method. Dentin is the most reliable substrate followed by composite.

## Background

Continuous modification of zirconia composition paved the way for the introduction of high translucent zirconia but these modifications can negatively affect the material’s strength and stability [1–6]. Monolithic zirconia exhibits supreme fracture strength and resistance compared to traditional porcelain-veneered restorations and other ceramic categories [7–9]. The evolving generations of monolithic zirconia as described by Cesar et al. [3] are 3Y-TZPs with low alumina content, 4Y- and 5Y-partially stabilized zirconia, Partially stabilized zirconia with above 5 mol% of yttria, color gradient, and composition-gradient zirconia (multilayered).

Adhesively cemented restorations show enhanced marginal adaptation, improved retention, and a higher capacity to resist microleakage compared to conventional cementation techniques [10, 11]. Intaglio zirconia surface conditioning is crucial for enhancing adhesion to resin cements due to its inherent non-silica composition, which limits traditional bonding methods [12, 13]. Methods like sandblasting, laser application, and chemical primers are commonly employed [14].

Air-borne particle abrasion provided a durable bonding to zirconia through increasing its surface energy, wettability, roughness, and the appearance of hydroxyl group that will facilitate bonding with the primer and the cement [14, 15]. However, Air-borne particle abrasion of zirconia provide a phase transformation where tetragonal phase transformed to monoclinic phase [16]. Comparing sandblasting with 120 μm alumina particles to 50 μm alumina particles, the results showed 15.8% and 6.1% monoclinic phase respectively [16]. Increased monoclinic content could be a factor in the material deterioration (microcracking and strength loss) [16, 17]. Therefore a strict protocol was introduced by Özcan (2013) to avoid weakening effect of air abrasion [18].

Chemical primers, particularly those containing phosphate ester monomers like MDP, significantly improve adhesion by forming chemical bonds with zirconia and resin cements [19–22].

Water storage and thermal cycling are key methods for assessing the durability of dental materials [23]. Water storage can weaken resin matrices and bonding through hydrolysis [24], while thermal cycling simulates oral temperature variations, inducing thermal stresses that lead to crack propagation and reduced material strength [25, 26]. Low temperature degradation (LTD) in zirconia occurs when the material transitions from the tetragonal to monoclinic phase under warm or humid conditions, leading to significant reduction in mechanical properties [27–29].

Many previous studies were conducted on different zirconia surface treatment and its effect on bonding strength [22, 30–40]. However, nearly no studies compared the bonding durability between different substrates and super translucent multilayered monolithic zirconia after different surface treatments. Furthermore, no research has reported the influence of the interaction of the two parameters on shear bond strength. As a result, the current study assessed the effects of various substrates and zirconia surface treatments, as well as their interactions, on the shear bond strength of super transparent multilayered monolithic zirconia.

The null hypotheses of the present study were that.

1-Application of universal primer will increase bond strength to zirconia regardless of sandblasting effect.

2- The bonded substrate will not affect the shear bond strength.

## Methods

### Materials

Materials used in this study are showed in Table 1.

**Table 1:** Showing materials used in this study

Materials	Product name	Composition	Manufacture	Lot number
Yttria partial stabilized zirconia	Katana zirconia super translucent multilayered (STML)	ZrO2, Y2O (4.8 mol%)	Kuraray Noritake Dental, Miyoshi-cho, Miyoshi, Aichi 470 − 0293, Japan	EIVIY
50 μm Al_2_O_3_	Shera Aluminum oxide 50 μm	99.7% aluminum oxide	Shera Werkstoff- Technologie, Germany	1,799,872
Universal primer	Monobond N	Silane methacrylate, phosphoric methacrylate and sulfide methacrylate	IvoclarVivadent AG, Schaan, Liechtenstein	Z02XRS
Dual curing adhesive resin cement	Multilink automix	Barium glass, ytterbium trifluoride, Bis-EMA, HEMA, Bis-GMA, Si-Zr mixed oxide, barium aluminium fluorosilicate glass, UDMA, highly dispersed silicon dioxide the total content of inorganic filler is approx. 40 vol%. Particle size of inorganic fillers is 0.15–15.5 μm.	IvoclarVivadent AG, Liechtenstein	Z03N31
Dental adhesive	Multilink Primer A and B	Water, phosphonic acid acrylate, HEMA, sulfinate, p-Toluidine, methacrylate-modified polyacrylic acid	IvoclarVivadent	Primer A: Z039SD Primer B: Z03BXV
Resin modified glass ionomer	Riva light cure capsules	ionglassTM filler, made of a unique blend of different sizes of ultrafine highly reactive glass particles. The ionglassTM filler contains fluoride and strontium ions	SDI Limited, Bayswater Victoria 3153, Australia.	J2204045EA
Composite	Brilliant EverGlow	Methacrylate-based matrix, silica and glass ceramic fillers, silane coupling agents, photo-initiators, and pigments	Coltene Holding AG Dübelstrasse 6 CH-9450 Altstätten Switzerland.	M55281

### Specimens preparation

**Fig. 1:**
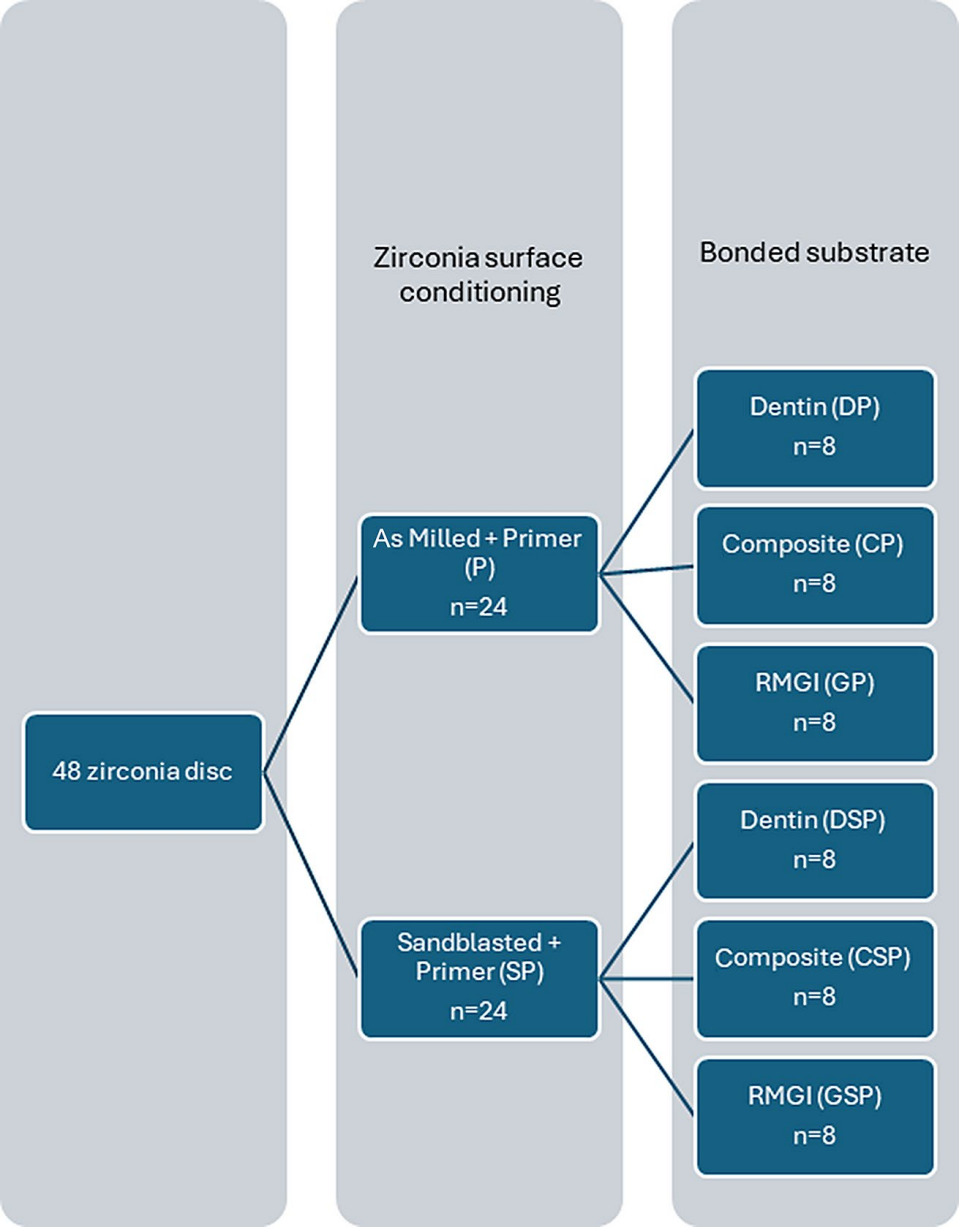
Illustrating grouping of specimens

Based on several published studies [22, 30–40] and using G power program version 3.1.9.7 to calculate sample size based on effect size of 1.39, using 2-tailed test, α error = 0.05 and power = 80.0%, the total calculated sample size was 8 in each group. Grouping of specimens is illustrated in (Fig. 1).

#### Zirconia discs fabrication

Zirconia discs were designed (8 × 3 in dimensions) and dry milled using 5-axis milling machine (Ceramill Motion 2, Amman Girrbach AG, Herrschaftswiesen, Austria) from a super translucent multilayered zirconia blank (Katana Zirconia STML, Kuraray Noritake Dental). Discs were sintered using high temperature furnace Ceramill Therm (Amman Girrbach AG, Herrschaftswiesen, Austria) according to the manufacturer’s specifications. A slot was made on the outer margin of the untreated surface using a straight handpiece.

#### Composite discs fabrication

Sixteen composite discs were made by using a Teflon mold that was fabricated with apertures in the needed dimension (8 × 3 mm). After application of composite (Brilliant EverGlow, coltene, Switzerland), curing was done for 40 s (liteQ LD-107, MONITEX, Taiwan) for both upper and lower surfaces. Discs were removed from the mold and finished. The untreated surface was marked with a slot on the outer margin by using straight handpiece.

#### Resin modified glass ionomer (RMGI) discs fabrication

RMGI (Riva light cure capsules, SDI Limited, Australia) discs were fabricated in a similar method described for composite disc fabrication.

### Dentin samples fabrication

Molars extracted from patients in good health because of periodontal conditions were gathered in accordance with guidelines accepted by the ethical committee at Mansoura University (see Appendix B). The dental patients involved were attending the outpatient clinic at the Faculty of Dentistry for dental care. After removing soft tissue remnants with a hand scaler (Goldman, Illinois, USA), the teeth were disinfected using a 1:10 dilution of 5.25% sodium hypochlorite (Clorox bleach, Clorox Co., Cairo, Egypt) within seven days. The gathered teeth were cleaned using a rubber cup and a fine pumice-water slurry. A total of sixteen molars were selected, all of which were free from cracks, carious lesions, and microfractures. Finally the collected teeth were kept in water that had been distilled at room temperature throughout the duration of the study, with the water being changed weekly to inhibit bacterial growth.

All molars were fixed vertically in acrylic resin blocks for easy handling. A diamond saw was used to perform standardized tooth preparation. The teeth were cut axially, and the coronal tooth portion 4.0 mm occlusal to the cementoenamel junction was cut. Dentin was exposed in the center with peripheral enamel.

### Specimen’s fabrication

### Zirconia discs surface treatment

All 48 zirconia discs were divided into two main groups (*n* = 24) based on surface treatment either original milled or after air-abrasion.

### Group (P): as milled and primer application

**Fig. 2:**
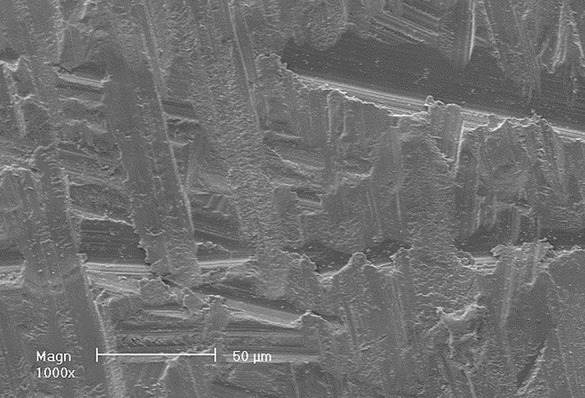
Representative SEM micrograph showing intaglio surface of original milled zirconia disc. Magnification X 1000

The intaglio surfaces of 24 zirconia discs were used as milled. Scanning electron microscope (SEM) imaging was made to examine zirconia samples as milled prior to primer application (Fig. 2).

A thin coat of Monobond N (IvoclarVivadent, Lot Z02XRS) was applied to intaglio surfaces using a microbrush. The primer was set to react for 60 s. A gentle flow of air was then used to disperse the left excess.

### Group (SP): airborne particle abrasion and primer application

**Fig. 3:**
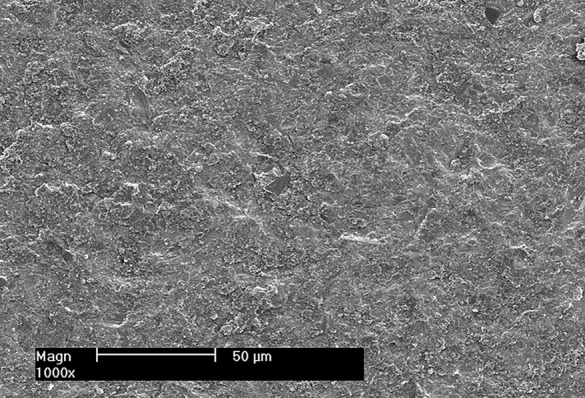
Representative SEM micrograph showing intaglio surface of airborne-particle abraded zirconia disc. Magnification X1000

1. Intaglio surfaces of the other 24 zirconia discs were marked and conditioned by air-abrasion with 50 μm Al_2_O_3_ (Shera Werkstoff- Technologie, Germany) at a pressure of 2 bar (Renfert basic professional sandblaster, Beckum, Germany) [18] till all the marker was wiped away. Intaglio surface of selected air abraded zirconia discs was examined using SEM at X 1000 (Fig. 3).
2. The zirconia discs finally cleaned with 100% alcohol in an ultrasonic cleaner for 10 min then dried.
3. A thin layer of Monobond N (IvoclarVivadent) was brushed onto the previously conditioned surface utilizing a microbrush. The substance was left to react for 60 s. Any residual excess was dissipated with a gentle stream of air.

#### Bonding of zirconia discs

Each main group was divided into three sub-groups (*n* = 8) based on the bonded substrate:

### Groups (CSP and CP): bonding to composite discs

Bonding procedures were carried out according to the manufacturer’s recommendations using adhesive resin cement (Multilink automix, Lot. Z03N31),

- Multilink automix resin cement was auto mixed by disposable auto mixing tip (working time was 3 min at room temperature) and applied on the conditioned bonding surfaces of discs.
- The disc/disc assembly was placed in a specifically built device that applied a continuous force (2 kg) to the assembly during cementation.
- Initial light curing was done for 2 s (liteQ LD-107, MONITEX, Taiwan) with intensity of 1600mW/cm on all four margins (mesial, distal, buccal, and palatal) then excess cement was removed by scaler and four margins were light cured again each for 20s.
- The cemented assembly remained for 5 min under the constant load.

### Groups (GSP and GP): Bonded to RMGI discs

RMGI discs were bonded to zirconia discs in a similar method that was described for composite samples.

### Groups (DSP and DP): Bonded to dentin samples

Self-curing Multilink Primer A/B was mixed, spread over the bonding surface of dentin with a micro brush and scrubbed for 30 s. Air was blown to remove excess bond till no mobile film was observed. Bonding of dentin to zirconia was done using Multilink automix resin cement with the same procedures used for previous test groups.

### Artificial aging

#### Water storage

Specimens were stored for 6 months in water bath at 37^o^C [14]after the cementation was completed by one hour.

#### Thermal cycling

Specimens were thermal cycled for 10,000 cycles to mimic one year clinically [41] in water between 5^o^C and 55^o^C. The specimens were kept in each temperature for 30 s while transfer time was 15 s. After thermal cycling SBS test was performed immediately.

### Shear bond strength test (SBS)

A universal testing machine (Instron 3345, USA) was used to apply compressive load at the zirconia/substrate interface at a crosshead speed 0.5 mm per minute. The load to failure was recorded in Newton. To obtain the SBS in MPa the failure load was divided by the bonded surface area.

### Mode of failure analysis

The mode of failure was examined by using a stereo microscope. Recorded failure patterns were adhesive, cohesive or mixed failure. Further investigation was done by using SEM imaging.

## Results

The collected data were subjected to statistical analyses with (SPSS) version 26. Normality test was carried out with Kolmogorov-Smirnov Test. All test groups did not deviate from normality. (Table 2)

Means failure loads (MPa) of all test groups were compared with a 2-factor ANOVA model (Table 3), including the following factors: surface treatment, substrate, and their interactions. The overall ANOVA F-test was significant (*P* = 0.000) indicating significant difference in mean shear bond strength of the two factors and their interaction as follow, surface treatment of zirconia (*P* = 0.000), bonded substrate (*P* = 0.000), and their interaction (*P* = 0.000).

Additional analyses with serial one-way (ANOVA)s were conducted to examine the influence of each factor separately. Surface treatment factor was significant, P value (0.042) (Table 4), and bonded substrate was highly significant P value (0.000) (Table 5).

**Fig. 4:**
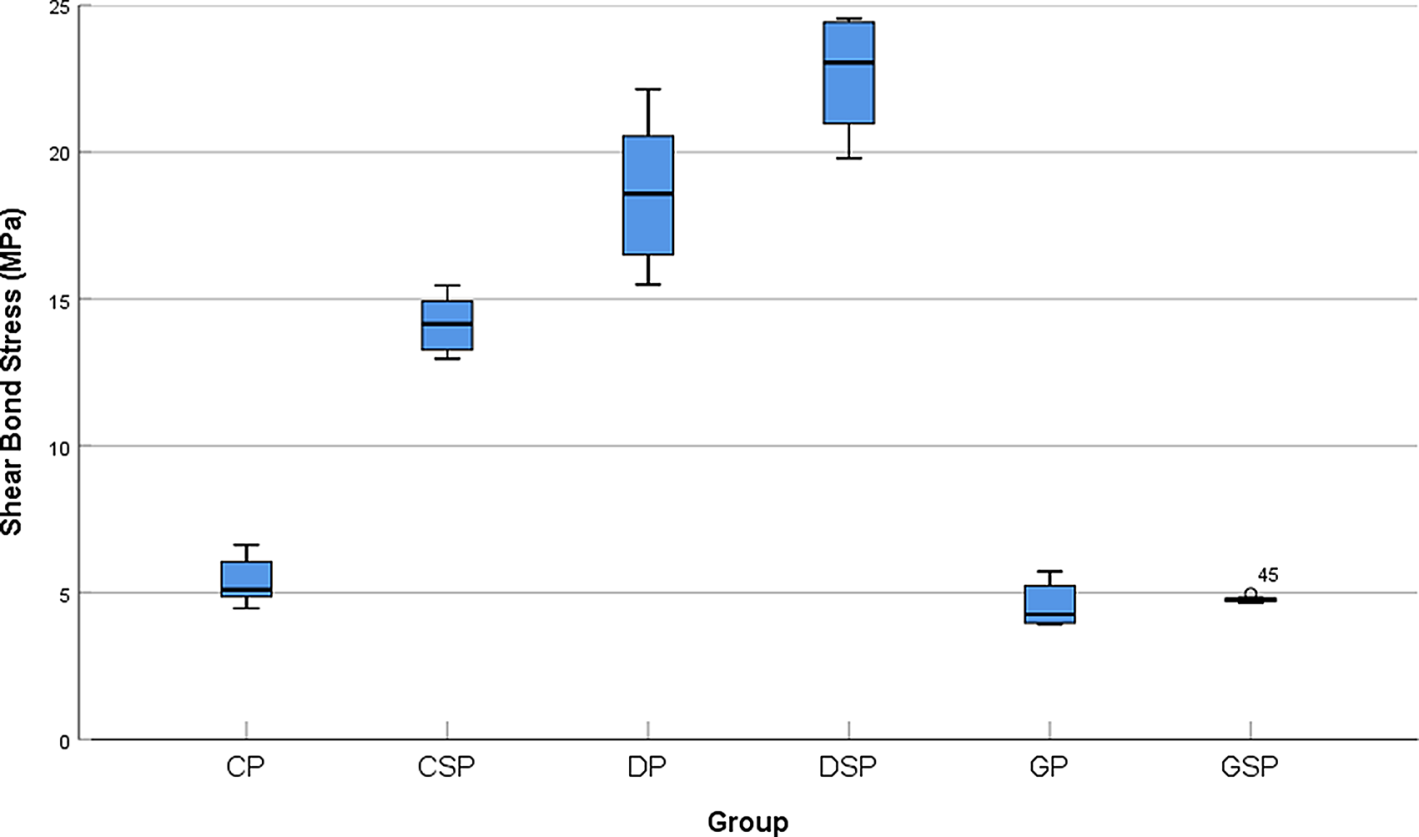
Box Plots showing shear bond strength in (MPa) of tested groups

Bonferroni Post Hoc test was employed for pairwise comparison among various test groups (Table 6). Regarding surface treatment of zirconia with the same substrate, there were statistically significant differences among the next test groups: (CP-CSP, *P* = 0.000), (DP-DSP, *P* = 0.000). Also, there was statistically significant differences between test groups (CSP-GSP, *P* = 0.000), (CSP-DSP, *P* = 0.000), (GSP-DSP, *P* = 0.000), (DP-CP, *P* = 0.000), (DP-GP, *P* = 0.000) when considering substrate at the same surface treatment. However, no statistically significant differences were found among the following test groups (GSP-GP, GSP-CP, CP-GP) (*P* > 0.05). Mean shear bond strength for all groups were demonstrated in box plots. (Fig. 4)

**Table 2:** Showing One-Sample Kolmogorov-Smirnov (normality) test

		CP	CSP	DP	DSP	GP	GSP
N		8	8	8	8	8	8
Normal Parameters^a, b^	Mean	5.3889350	14.1339700	18.6085663	22.6486850	4.5715225	4.7654650
	Std. Deviation	0.77529680	0.95174716	2.55610564	2.00611899	0.73353668	0.09307189
Most Extreme Differences	Absolute	0.234	0.142	0.196	0.241	0.244	0.233
	Positive	0.234	0.138	0.196	0.172	0.244	0.233
	Negative	-0.152	-0.142	-0.166	-0.241	-0.188	-0.117
Test Statistic		0.234	0.142	0.196	0.241	0.244	0.233
Asymp. Sig. (2-tailed)		.200^c, d^	.200^c, d^	.200^c, d^	.191^c^	.177^c^	.200^c, d^

**Table 3:** Showing two-way ANOVA test at different level of the study

Source	Type III Sum of Squares	df	Mean Square	F	*p*-value
Model	9053.279a	6	1508.880	717.840	0.000
Surface Treatment	224.609	1	224.609	106.856	0.000
Substrate	2126.717	2	1063.359	505.886	0.000
Surface Treatment x Substrate	146.734	2	73.367	34.904	0.000
Error	88.283	42	2.102		
Total	9141.562	48			

**Table 4:** Showing one-way ANOVA considering different surface treatments

	Sum of Squares	df	Mean Square	F	Sig.
Between Groups	224.609	1	224.609	4.375	0.042*
Within Groups	2361.734	46	51.342		
Total	2586.343	47			

(*) indicating statistically significant difference

**Table 5:** Showing one-way ANOVA considering different substrates

	Sum of Squares	df	Mean Square	F	Sig.
Between Groups	2126.717	2	1063.359	104.109	0.000*
Within Groups	459.626	45	10.214		
Total	2586.343	47			

(*) indicating statistically significant difference

**Table 6:** Showing results of Bonferroni Post Hoc test (*p* ≤ 0.05)

Groups	Mean ± SD	CP	CSP	DP	DSP	GP	GSP
CP	5.39 ± 0.77		0.000*	0.000*	0.000*	1.000	1.000
CSP	14.13 ± 0.95	0.000*		0.000*	0.000*	0.000*	0.000*
DP	18.61 ± 2.56	0.000*	0.000*		0.000*	0.000*	0.000*
DSP	22.65 ± 2.0	0.000*	0.000*	0.000*		0.000*	0.000*
GP	4.57 ± 0.73	1.000	0.000*	0.000*	0.000*		1.000
GSP	4.77 ± 0.09	1.000	0.000*	0.000*	0.000*	1.000	

C; Composite G; Resin modified glass ionomer D; Dentine

P; Primer application SP; Sandblast and Primer application

(*) indicating statistically significant difference

### Mode of failure analysis

**Fig. 5:**
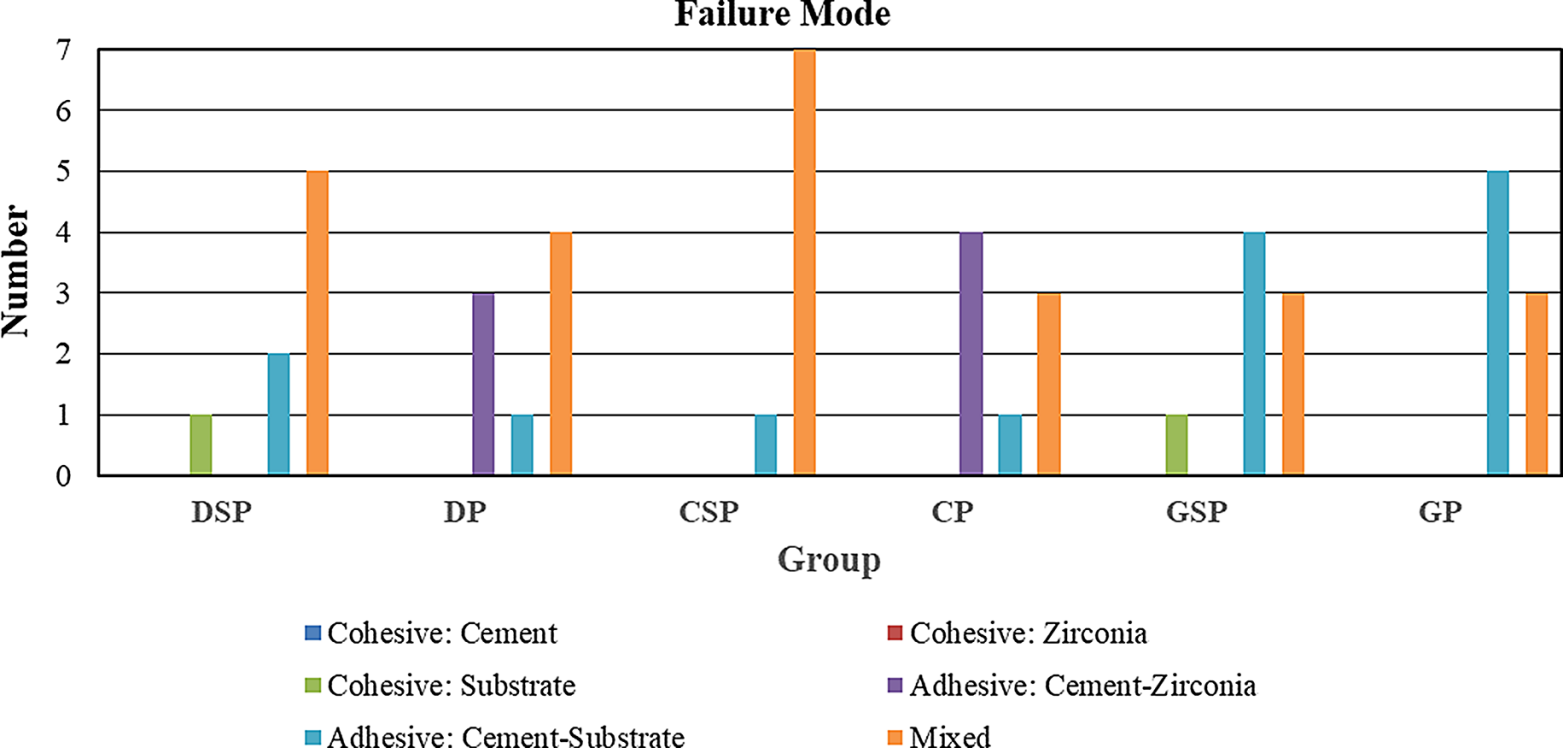
Showing different failure modes for all test groups

**Fig. 6:**
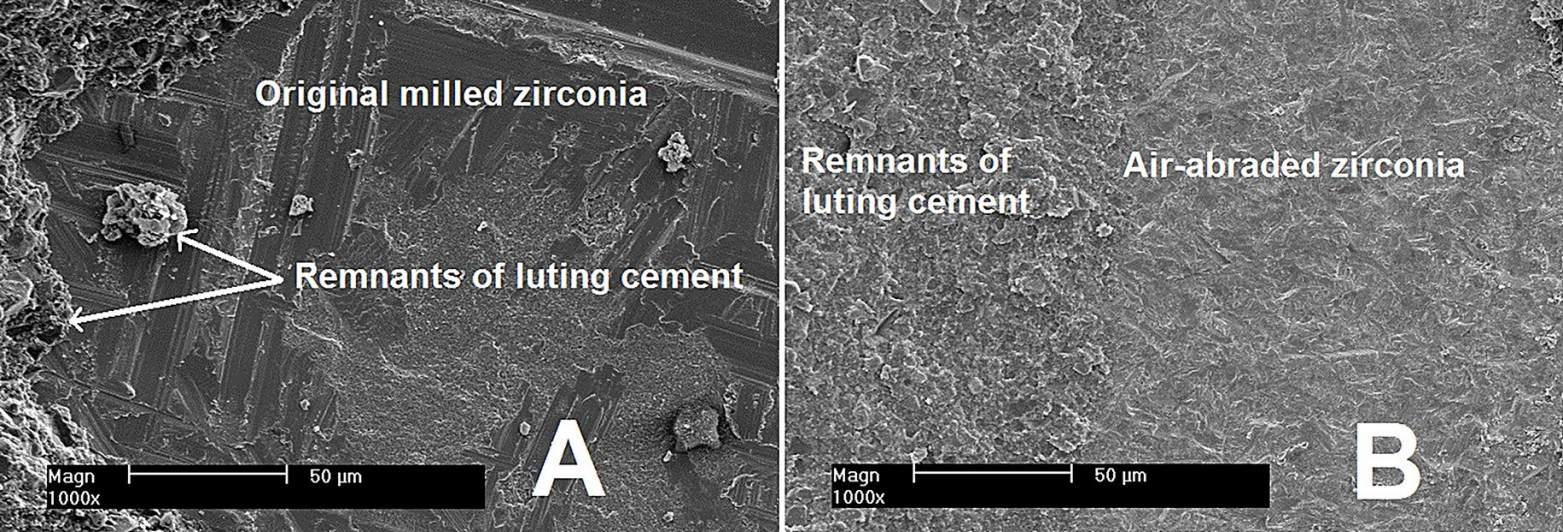
Representative SEM micrograph showing the intaglio surface of original milled zirconia (**A**) and air- abraded (**B**) after debonding with remnants of luting cement adhered to it. Magnification ×1000

**Fig. 7:**
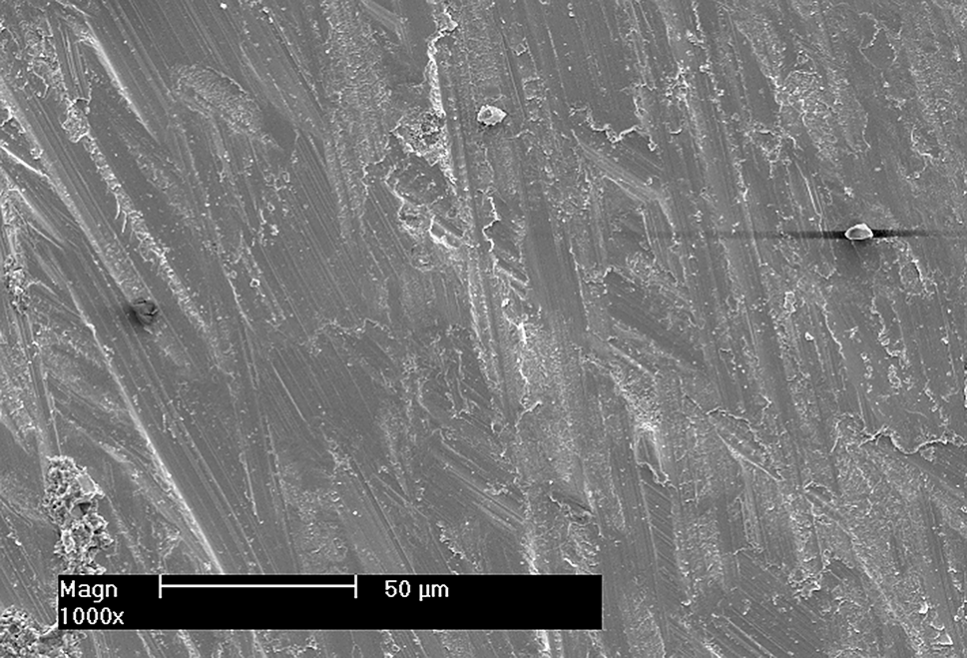
Representative SEM micrograph showing intaglio surface of original milled zirconia with adhesive failure mode. Magnification ×1000

After debonding of samples remnants of resin cement were still adhered either to zirconia surface or to the substrate surface. Failure modes were examined by using a stereo microscope. The most common failure mode was mixed (failure mode III) with 25 specimens, followed by adhesive (failure mode II) with 21 specimens then cohesive (failure mode I) with two specimens. Cohesive failure in the substrate was found in DSP and GSP groups. Adhesive failure at the zirconia-cement interface was found in DP and CP groups. Adhesive failure at substrate-cement interface was found in all test groups but predominantly in GSP and GP groups. Mixed failure was found in all test groups predominantly in CSP, DSP and DP groups (Fig. 5). Further analysis was done using SEM imaging for representative samples for each group at magnification X1000 (Fig. 6) and (Fig. 7).

### Discussion

This invitro study investigated the influence of surface conditioning of super translucent multilayered monolithic zirconia and the underlying substrate on the shear bond stress. Moreover, the study investigated possible correlation between zirconia surface treatment and type of substrate on bonding durability to zirconia.

The null hypothesis of the current study was universal primer application will increase bonding durability to zirconia regardless of surface treatment. While the bonded substrate would not have effect on the shear bond. However, results of this study rejected both parts of the hypothesis. Sandblasting with primer significantly increased bond strength than original milled zirconia with primer. Regarding the substrate, bonding of zirconia to dentin was significantly higher than bonding of zirconia to composite and RMGI.

To replicate clinical conditions, water storage for six months was included to simulate the effect of moisture. Subsequently, specimens were thermal cycled for 10,000 cycles, a procedure that Gale and Darvell [41] have suggested is roughly equivalent to one year clinically. The results proved that long-term water storage and thermal cycling negatively influenced bond strength due to factors such as water-induced deterioration of the bonding interface, mismatched coefficient of thermal expansion (between substrates, zirconia and resin cement), water sorption and hydrothermal degradation of the luting resin, deterioration of silanized surfaces and thermal stress due to thermocycling [42–45]. Thus the cumulative negative impact of these factors likely contributes to the observed reduction in bond strength. However, the aging regime did not lead to spontaneous failures.

Shear bond strength tests are generally easy to execute and may be done without expensive equipment. They imitate the forces produced by restorations during masticatory movements [46]. However stresses at the interface are not equally dispersed, there is a significant rate of mixed and cohesive failures, no specific information on failure processes is provided, and surface conditioning can considerably influence outcomes, introducing discrepancies [47].

A clinically acceptable minimum bond strength is recognized to fall within the range of 10–13 MPa [40]. Three test groups reported bond strength results which considered acceptable clinically DSP 22.7 ± 2 MPa, DP18.6 ± 2.3 MPa and CSP14.1 ± 1 MPa. On the other hand, the other three test groups CP 5.39 ± 0.8 MPa, GSP 4.77 ± 0.09 MPa and GP 4.57 ± 0.7 MPa showed bond strength results lower than the clinically acceptable bond strength range.

Research shows that air abrasion can compromise the integrity of high-strength ceramics, potentially reducing the clinical longevity of zirconia restorations, especially those with thin Sect. [16]. To address this issue, this study examined the bonding to zirconia both in its original milled state versus after undergoing air abrasion. The milling process may leave residual carbonaceous contamination, affecting surface wettability and bond stability [48]. In contrast, air abrasion with aluminum oxide enhances surface roughness and energy, improving bonding by increasing wettability and facilitating resin tag formation [14].

The universal primer in this study forms a chemical bond with both the adhesive resin cement by silane, and the conditioned zirconia surface through phosphoric acid methacrylate and sulfide methacrylate groups. The phosphate group in the acid monomer forms a chelation bond with the zirconia surface. The phosphoric acid ester or phosphate ester functional group reacts with the zirconium atoms (Zr) in the zirconia, creating a Zr-O-P bond. On the other side this group allows copolymerization between monomer included in the universal primer with the monomers of the resin cement. Moreover, according to the manufacturer, the silane coupling agent is included in universal primer, therefore bonding to adhesive resin cement was extra strengthen [19, 22, 49, 50].

Regarding the two surface treatments with the same substrate used in this study considering composite resin, there was a significant difference between group CSP (14.13 ± 0.95 MPa) and group CP (5.39 ± 0.8 MPa) groups with higher mean value for group CSP.

Similarly, Yi et al. (2015) [51] and Barragan et al. (2014) [52] revealed that universal primer application after air abrasion significantly increased bond strength (8.93 ± 3.13 MPa) and (15.3 ± 6.4 MPa) compared to application of universal primer without sandblasting (3.91 ± 0.72 MPa) and (7.7 ± 3.6 MPa).

Regarding dentine substrate, there was significant difference between group DSP (22.65 ± 2.0 MPa) and group (DP 18.61 ± 2.6 MPa) with the higher bond strength reported for group DSP. Alves et al. (2016) [30], zhang and degrange (2010) [31] and Afrasiabi et al. (2018) [32] found that using 10-MDP containing primer provided a durable bonding at zirconia /dentin interface.

Regarding RMGI substrate, air abrasion did not enhance the bond strength of zirconia/RMGI assembly. There was no significant difference between test groups GSP (4.77 ± 0.09 MPa) and GP (4.57 ± 0.7 MPa).

Considering the three substrates regardless of zirconia surface treatment, dentine showed the highest bond strength followed by composite resin. RMGI came at last. These results could be explained by the fact that both composite resin and RMGI discs were bonded without any surface treatment. On the other hand, dentin discs were treated by conditioning with mix of self-adhesive A&B primer that allowed the formation of micromechanical bond with dentine. This micromechanical bonding was nearly absent in the case of composite resin and RMGI discs.

Additionally, both GSP and GP groups showed the lowest mean shear bond strength values between test groups. Many factors could explain these results. Such as the low bond capability between resin cement and RMGI [53]. Also, inherent low mechanical properties of RMGI compared to composite resin and dentin knowing that flexural strength of RMGI ranges from 50 to 60 MPa, while flexural strength of dentin and composite are 245–280 MPa, 85–110 MPa respectively [46]. Additionally, RMGI’s sensitivity to water, water infiltration disrupts the polymer network, reducing strength and increasing solubility [54]. Finally, differences in the coefficient of thermal expansion between RMGI (10.2–11.4 ppm) and other materials like dentin (11.4 ppm), composite (14.50 ppm), and zirconia (9.8 ppm) might contributed to varying thermal stresses during thermal cycling.

The mode of failure of GSP and GP groups was mainly adhesive at the cement-substrate interface which confirmed the poor bond between resin cement and RMGI. These results are in accordance with several published literatures tested RMGI as a cement and it was concluded that RMGI had a weak bond with zirconia [55, 56].

Results of this study have proven the interaction between zirconia surface treatment and the bonded substrate. The combination between dentine substrate and surface treating zirconia with sandblasting then primer application gave the best result. While glass ionomer substrate along with treating zirconia with primer gave the worst result.

Mixed failures (Mode III), involving both adhesive and cohesive failures, were predominant in the DSP and CSP groups, indicating that both the bonding interface and the material contributed to the failure. This suggests variability in the effectiveness of surface treatments and bonding processes. In contrast, adhesive failures (Mode II) were more common at the cement-zirconia interface in the DP and CP groups, and at the cement-substrate interface in the GSP and GP groups, highlighting issues with the bonding interface. Cohesive failures (Mode I) were rare, primarily occurring in the DSP and GSP groups as substrate fractures, indicating a strong bond but potential weaknesses in the substrate material under stress.

When bonding zirconia to dentin surface, both surface treatments are accepted whether sandblasting then priming or priming only. However, a significant difference was reported between DSP and DP with a higher bond strength value for DSP but still DP had higher mean shear bond strength than composite groups and RMGI groups. While bonding zirconia to composite substrate, it is recommended to treat zirconia with sandblasting then primer rather that primer only. However, RMGI is not recommended to be used as a substrate material.

limited number of thermal cycles, absence of cyclic loading fatigue, and storage was done in water only not saliva and other beverages were the limitations of the current study.

## Conclusions

Within the limitations of this invitro study, the following conclusions were reached:

1. Zirconia surface treatment, bonded substrate and their interaction affected the shear bond strength.
2. Regarding substrate, dentine is the most favorable one followed by composite, but RMGI is not recommended.
3. Regarding surface treatment, sandblasting followed by universal primer application enhanced bonding to zirconia compared to original milled and universal primer application.

## Data Availability

The data sets used and/or analyzed during the current study are available from the corresponding author upon reasonable request.

## Abbreviations

RMGI: Resin modified glass ionomer
SEM: Scanning electron microscope
EDM: Electrical discharge machining
MDP: Methacryloyloxydecyl Dihydrogen Phosphate
Y-PSZs: Yttria-partially stabilized zirconia
LTD: Low temperature degradation
CAD/CAM: Computer aided design; computer aided manufacture
SPSS: Social Package for Statistical Science
